# Associations of body mass index and waist circumference with all cause mortality in the oldest old with cognitive impairment: a prospective cohort study

**DOI:** 10.3389/fnut.2025.1561909

**Published:** 2025-07-02

**Authors:** Xiaopeng Li, Peng Zhao, Mei Zhao

**Affiliations:** ^1^Department of Neurology, The First Affiliated Hospital of Henan University, Kaifeng, China; ^2^Lankao Hospital of Traditional Chinese Medicine, Kaifeng, China; ^3^Department of Neurology, The First Affiliated Hospital of Nanchang University, Nanchang, China; ^4^Institute of Neurology, Jiangxi Academy of Clinical Medical Science, The First Affiliated Hospital, Jiangxi Medical College, Nanchang University, Nanchang, China; ^5^Rare Disease Center, The First Affiliated Hospital, Jiangxi Medical College, Nanchang University, Nanchang, China; ^6^Key Laboratory of Rare Neurological Diseases of Jiangxi Provincial Health Commission, Jiangxi Medical College, Nanchang University, Nanchang, China

**Keywords:** body mass index, waist circumference, all cause mortality, the oldest old, cognitive impairment

## Abstract

**Importance:**

In clinical practice, reducing body mass index (BMI), and waist circumference (WC) is a crucial treatment target for minimizing health risks. However, the association patterns between BMI, WC, and all cause mortality in cognitively impaired older adults remain unknown.

**Objective:**

To investigate the association patterns between body mass index (BMI), waist circumference (WC), and all cause mortality among cognitively impaired oldest old.

**Design, setting, and participants:**

The cognitively impaired oldest old from the Chinese Longitudinal Healthy Longevity Survey (CLHLS) in the 2011–2014 wave was included. A restricted cubic spline based on Cox proportional hazards model was used to examine the association patterns.

**Exposures:**

The global cognitive function of participants was assessed by the Minimum-Mental State Examination.

**Main outcomes and measures:**

All cause mortality was the outcome.

**Results:**

A total of 2,124 participants (1,522 females [71.7%]) were included in this study and 1,071 (50.4%) deaths were documented. We found J shaped association between levels of BMI and all cause mortality in cognitively impaired oldest old, with low BMI levels associated with increased mortality risk whereas high BMI levels associated with reduced mortality risk. Compared to BMI in quartile 4, the multivariable adjusted hazards ratios of all cause mortality were 1.53 (95% confidence interval, 1.28 to 1.83), 1.36 (1.13 to 1.64), and 1.31 (1.09 to 1.57), respectively, for BMI in quartiles 1, 2 and 3. We also observed a linear association between levels of WC and all cause mortality in the cognitively impaired oldest old, with low levels associated with high mortality risk and high levels associated with low mortality risk. In contrast with lower WC (quartile 1), the hazard ratios for all cause mortality were 0.82 (0.70 to 0.97) for quartile 2, 0.77 (0.65 to 0.91) for quartile 3, and 0.70 (0.59 to 0.83) for quartile 4, respectively. Joint association analyses revealed that participants in the highest quartile of BMI and the highest quartile of WC had the lowest mortality risk.

**Conclusions and relevance:**

Among the cognitively impaired oldest old, we found a J shaped association between BMI and all-cause mortality, and a linear association between WC and all-cause mortality, with increased levels of each associated with reduced mortality risk. Contrary to clinical practices that aim to reduce BMI and WC to minimize health risks, this study emphasizes the importance of maintaining higher BMI and WC levels in cognitively impaired oldest old.

## Introduction

With the aging population growing in China, older adults aged 80 years or more will reach 115 million in 2050 (1). Mild cognitive impairment prevalence in China for older adults has been estimated to be 15.5% (2). This might suggest that a larger population of cognitively impaired oldest old needs care, particularly those facing challenges in daily life, such as higher rates of falls and hospitalizations, as well as those at increased risk for dementia (3–5). High levels of Body mass index (BMI) and waist circumference (WC), both indicators of abdominal obesity, have been suggested to be associated with increased mortality risk in the general population (6). Adults with higher WC are also at increased adverse health risks (7–9). In clinical practice, reducing WC is a crucial treatment target for minimizing health risks in both men and women (10). Further, recent studies suggest that nearly one in four older adults (aged 65 and over) are either malnourished or at risk of malnutrition, particularly undernutrition, which is often associated with reduced BMI (11, 12). With the continued demographic shift toward an aging population, this number is anticipated to increase substantially over time (11). Undernutrition has been associated with negative outcomes such as frailty, cognitive decline, reduced quality of life, and increased risk of death (11, 13–15). However, to our knowledge, the association patterns between higher or lower levels of BMI, WC, and all cause mortality among older adults with cognitive impairment remains unknown. The only existing evidence was sourced from two studies focused on dementia. One cohort study with patients diagnosed with incident dementia reported that higher BMI at the time of dementia diagnosis was associated with lower mortality risk (16). Another cohort study found a negative relation between obesity and dementia-related mortality (17). Furthermore, the specific association patterns between BMI and mortality using restricted cubic splines were not examined in these two studies.

To fill this critical gap, using data from the Chinese Longitudinal Healthy Longevity Survey (CLHLS) 2011–2014, we aimed to examine the two indicators of abdominal obesity, BMI and WC, in relation to all cause mortality among the cognitively impaired oldest old.

## Methods

### Study population

The CLHLS is a nationwide survey covering about 85% of China’s population, carried out in randomly chosen areas within 22 provinces (18, 19) It employed a targeted random sampling method to ensure gender balance and adequate representation of the oldest-old (aged 80 and above) (18). Over the period 1998–2018, the CLHLS completed eight waves of surveys in China, employing survey instruments aligned with international standards. Researchers have systematically assessed various aspects of CLHLS data quality—such as mortality rate, and reliability and validity of major health measures—and found them to be generally satisfactory compared with other large-scale aging studies (18). Rigorous evaluations have confirmed that the CLHLS data are of reasonably high quality (20, 21). The percentage of missing data is relatively low, ranging from 1 to 3% (18). The survey was approved by the Medical Research Ethics Committee of Peking University. All participants provided written informed consent.

A total of 9,765 older adults participated in the 2011 wave of CLHLS. The global cognitive function of participants was assessed using the Chinese version of the Mini-Mental State Examination (MMSE), which had been culturally adapted and translated based on the international standard of the MMSE questionnaire and had been tested in the pilot survey (22). The adaptation aimed to align the questions with the participants’ cultural and socioeconomic backgrounds, making them easier to comprehend and respond to Yi and Vaupel (22). The MMSE consists of 24 items with a total score ranging from 0 to 30, covering five domains: orientation, registration, calculation, attention, recall, and language (18). The reliability of MMSE in CLHLS reaches high (Cronbach’s a = 0.96) and has been validated in other studies (18, 23–25). In the CLHLS dataset, raw MMSE scores were not directly adjusted for age or educational level. Instead, age and educational level were included as covariates in our regression models to control for their potential confounding effects. Following Zeng et al. (18) procedure, we divided participants into cognitive impairment (0–22) and normal cognitive function (23–28) by the score of MMSE. In all items, the missing values were less than 1.2% except the item of the drawing (16.3%). We excluded participants with missing values in items. Given the average age of 85.8 years, the low education level, and the eyesight of the oldest old, we considered these contributing to the difficulty of the drawing task. Thus, we treat the missing value in the item of the drawing as a score of 0. Finally, 3,034 participants were classified as having cognitive impairment. In supplement analysis, we also excluded participants with missing values in the item of the drawing and ran complete case analyses.

In the present study, we included 3,034 participants at baseline in the 2011 wave. All participants were followed up until the 2014 wave for all cause mortality. We excluded participants who were lost in follow-up (228), those with missing values in the measurement of body weight, body height, or waist circumference (498), those with three or more missing values in covariates (122), those with missing values in mortality date or with incorrect mortality date (62). Therefore, a total of 2,124 participants were included in the current analysis.

### Assessment of BMI and WC

In terms of physical examination, the body weight, height, and WC were measured using a standardized protocol by trained medical members (26). During the 2011 wave, body weight was recorded to the nearest 1 kilogram, whereas height and waist circumference were measured to the nearest 1 centimeter (26, 27). We calculated body mass index as body weight (kg) divided by height squared (m^2^). BMI was categorized into four quantiles: quantile 1 (BMI ≤ 17.8 kg/m^2^), quantile 2 (17.8 kg/m^2^ < BMI ≤ 19.8 kg/m^2^), quantile 3 (19.8 kg/m^2^ < BMI ≤ 22.3 kg/m^2^), and quantile 4 (BMI > 22.3 kg/m^2^). Waist circumference (WC) was similarly divided into four quantiles: quantile 1 (WC ≤ 70 cm), quantile 2 (70 cm < WC ≤ 78 cm), quantile 3 (78 cm < WC ≤ 85 cm), and quantile 4 (WC > 85 cm).

### Assessment of mortality

The mortality status was identified via interviewing family members if participants died during three years of follow-up until 2014 in CLHLS. The date of death was collected from participants’ close relatives, the residents’ committee, or according to official death certificates (28).

### Statistical analyses

Baseline continuous variable characteristics were expressed as means and standard deviations, whereas categorical variables were described using frequencies and percentages. To examine the dose–response relationship between BMI, WC, and all-cause mortality, the restricted cubic spline based on Cox proportional hazards models was used to estimate hazard ratios and 95% confidence intervals (29). Three knots were selected: the 5th, 50th, and 95th percentiles. We also conducted Cox proportional hazards models to estimate the hazard ratios and 95% confidence intervals for categories of BMI and WC in relation to all cause mortality. We checked the proportional hazards assumption by plotting parallel Kaplan–Meier curves and did not find evidence of violation. Several covariates have missing values and missing data in covariates were less than 2% except for ethnicity (7.8%). We did multiple imputations for these missing data (30).

The ethnicity variable was categorized as Han and non-Han. Education level was classified based on whether individuals had one year or more of schooling. Living patterns were determined by the presence or absence of family members within the household. Other covariates were also recorded including age, sex, residence, marriage, smoking, drinking, economic independence, regular exercise, hypertension, heart disease, diabetes, stroke, and adequate medication. The details of covariates were also depicted in previous studies (18, 25, 27, 31–33).

We adjusted for age (not adjusted in subgroup analysis by age), sex (not adjusted in subgroup analysis by sex), ethnicity (Han, non-Han), and residence (urban, rural) in model 1. We further adjusted for education level (1 year or more of schooling or not), marriage (in marriage, not in marriage), smoking (current smoking or not), drinking (current drinking or not), economic independence (yes or no), living pattern (living with family member or not) in model 2. We additionally adjusted for regular exercise (yes or no), hypertension (yes or no), diabetes (yes or no), heart disease (yes or no), stroke (yes or no), and adequate medication (yes or no).

All statistical analyses were conducted with SAS 9.4 (SAS Institute, Cary, NC) and R 4.3.2 software (34, 35). Two-sided *p* < 0.05 was considered as statistically significant.

## Results

### Baseline characteristics

A total of 2,124 participants (1,522 females [71.7%]) were included in the present study (Table 1) and 1,071 (50.4%) deaths were recorded. The mean age of participants was 93.2 years (SD: 9.1), with a mean BMI of 21.2 kg/m^2^ (SD: 15.7) and a waist circumference of 78.3 cm (SD: 13.3). Compared to the survival, those deaths were more likely to be older than 90 years, and were less likely to be in marriage and involved in regular exercise.

**Table 1:** Baseline characteristics of participants by mortality status at the end of follow-up*.

Characteristics	Total	Survival	Death
No of participants	2,124	1,053	1,071
BMI	21.2 (15.7)	21.5 (16.0)	20.9 (15.4)
WC	78.3 (13.3)	79.7 (12.7)	77.0 (13.8)
Women	1,522 (71.7%)	779 (74.0)	743 (69.4)
Age			
< 90 years	677 (31.9)	466 (44.3)	211 (19.7)
≥ 90 years	1,447 (68.1)	587 (55.8)	860 (80.3)
Ethnicity			
Han	1983 (93.4)	972 (92.3)	1,011 (94.4)
Non-Han	141 (6.6)	81 (7.7)	60 (5.6)
Residence			
Urban	283 (13.3)	132 (12.5)	151 (14.1)
Rural	1841 (86.7)	921 (87.5)	920 (85.9)
Education level	395 (18.6)	184 (17.5)	211 (19.7)
Marriage			
In marriage	333 (15.7)	212 (20.1)	121 (11.3)
Not in marriage	1791 (84.3)	841 (79.9)	950 (88.7)
Smoking	257 (12.1)	128 (12.2)	129 (12.0)
Drinking	269 (12.7)	132 (12.5)	137 (12.8)
Economic independence	195 (9.2)	109 (10.4)	86 (8.0)
Living pattern	1768 (83.2)	855 (81.2)	913 (85.3)
Regular exercise	458 (21.6)	286 (27.2)	172 (16.1)
Hypertension	556 (26.2)	303 (28.8)	253 (23.6)
Diabetes	49 (2.3)	27 (2.6)	22 (2.1)
Heart disease	231 (10.9)	112 (10.6)	119 (11.1)
Stroke	173 (8.2)	87 (8.3)	86 (8.0)
Adequate medication	1952 (91.9)	969 (92.0)	983 (91.8)

*Values are shown as numbers (percentages). Education indicates 1 year or more of schooling. Smoking and drinking represents the current state of smoking and drinking. The living pattern indicates living with family members.

### Relations of BMI and WC with all cause mortality

**Figure 1:**
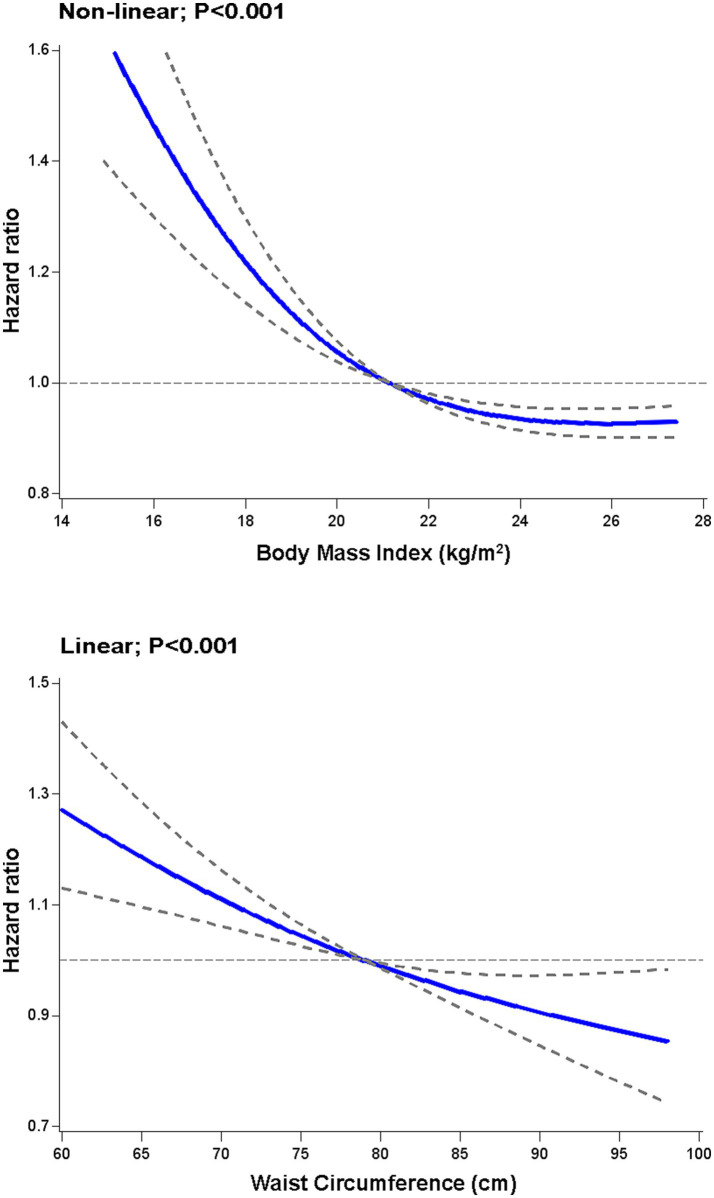
Restricted cubic spline analysis of body mass index and waist circumference in relation to all cause mortality in the oldest old with cognitive impairment. The hazard ratios were calculated for all cause mortality according to levels of body mass index and waist circumference among cognitively impaired oldest old. Associations were examined using restricted cubic spline based on Cox proportional hazards models. Risk estimates were adjusted for age, sex, ethnicity, residence, education level, marriage, smoking, drinking, economic independence, living pattern, regular exercise, hypertension, diabetes, heart disease, stroke, and adequate medication. The units for BMI and WC are kg/m^2^ and cm, respectively. The solid line is the estimated hazard ratio and the dashed line indicates 95% confidence intervals. *p* values for overall associations were all <0.001.

Among the cognitively impaired oldest old, the association between levels of BMI and all cause mortality was J shaped (*p* < 0.001); low BMI levels were associated with increased mortality risk whereas high BMI levels were associated with reduced mortality risk (Figure 1). Compared to participants with BMI in quartile 4, the multivariable adjusted hazards ratios of all cause mortality were 1.53 (95% confidence interval, 1.28 to 1.83), 1.36 (1.13 to 1.64), and 1.31 (1.09 to 1.57), respectively, for those with BMI in quartile 1, quartile 2 and quartile 3 (Table 2). When we excluded participants with missing values in the item of the drawing, the association patterns were similar (Supplementary Figure 2).

**Table 2:** Associations between BMI, WC, and all cause mortality in the oldest old with cognitive impairment*.

Variable	No (%) deaths	Model 1	Model 2	Model 3
BMI				
Quartile 1	331 (56.1)	1.52 (1.28 to 1.81)	1.52 (1.28 to 1.81)	1.53 (1.28 to 1.83)
Quartile 2	260 (53.0)	1.34 (1.12 to 1.61)	1.34 (1.12 to 1.61)	1.36 (1.13 to 1.64)
Quartile 3	266 (51.0)	1.26 (1.05 to 1.51)	1.27 (1.06 to 1.53)	1.31 (1.09 to 1.57)
Quartile 4	214 (41.1)	1.00	1.00	1.00
WC				
Quartile 1	321 (57.5)	1.00	1.00	1.00
Quartile 2	271 (50.3)	0.81 (0.69 to 0.96)	0.82 (0.69 to 0.96)	0.82 (0.70 to 0.97)
Quartile 3	246 (49.2)	0.76 (0.64 to 0.90)	0.76 (0.64 to 0.90)	0.77 (0.65 to 0.91)
Quartile 4	233 (44.2)	0.69 (0.58 to 0.82)	0.69 (0.58 to 0.82)	0.70 (0.59 to 0.83)

*BMI: body mass index; WC: waist circumference. Model 1 was adjusted for age, sex, ethnicity, and residence. Model 2 was additionally adjusted for education level, marriage, smoking, drinking, economic independence, and living pattern. Model 3 was further adjusted for regular exercise, hypertension, diabetes, heart disease, stroke, and adequate medication.

A linear association between levels of WC and all cause mortality (*p* < 0.001) was found in the cognitively impaired oldest old, with low levels associated with high mortality risk and high levels associated with low mortality risk (Figure 1). In contrast with lower WC (quartile 1), the multivariable adjusted hazards ratios of all cause mortality were 0.82 (0.70 to 0.97) for quartile 2, 0.77 (0.65 to 0.91) for quartile 3, and 0.70 (0.59 to 0.83) for quartile 4, respectively (Table 2). In subgroup analyses by sex, the multivariable adjusted hazard ratios for all cause mortality for BMI in quartiles 1, 2, and 3 were 1.49 (1.19–1.85), 1.34 (1.06–1.69), and 1.30 (1.04–1.64) for women, and 1.61 (1.18–2.19), 1.30 (0.95–1.78), and 1.26 (0.93–1.71) for men, respectively (Table 3). For WC, women in quartiles 2, 3, and 4 had multivariable-adjusted hazard ratios of all cause mortality of 0.84 (0.70–1.02), 0.77 (0.63–0.94), and 0.70 (0.57–0.86), respectively, while the corresponding values for men were 0.76 (0.55–1.07), 0.75 (0.54–1.05), and 0.72 (0.52–1.01) (Table 3). In terms of BMI and WC, *p* values for interaction by sex were 0.83 and 0.44, respectively. We also did not find significant interactions by baseline age and history of cardiovascular disease for BMI and WC (Table 3; Supplementary Table S1). Our main results mostly remained in subgroup analyses.

**Table 3:** Hazard ratios of all cause mortality according to BMI and WC stratified by sex and baseline age in the oldest old with cognitive impairment*.

Variable	No (%) deaths	Model 1	Model 2	Model 3
Women				
BMI				
Quartile 1	249 (54.4)	1.52 (1.22 to 1.89)	1.50 (1.21 to 1.87)	1.49 (1.19 to 1.85)
Quartile 2	185 (51.4)	1.38 (1.10 to 1.73)	1.36 (1.08 to 1.71)	1.34 (1.06 to 1.69)
Quartile 3	184 (49.6)	1.30 (1.04 to 1.64)	1.30 (1.03 to 1.63)	1.30 (1.04 to 1.64)
Quartile 4	125 (37.5)	1.00	1.00	1.00
WC				
Quartile 1	263 (56.6)	1.00	1.00	1.00
Quartile 2	181 (49.2)	0.82 (0.68 to 1.00)	0.83 (0.69 to 1.01)	0.84 (0.70 to 1.02)
Quartile 3	154 (46.5)	0.75 (0.62 to 0.92)	0.74 (0.61 to 0.91)	0.77 (0.63 to 0.94)
Quartile 4	145 (40.5)	0.66 (0.54 to 0.81)	0.67 (0.55 to 0.82)	0.70 (0.57 to 0.86)
Men				
BMI				
Quartile 1	82 (62.1)	1.56 (1.16 to 2.11)	1.59 (1.17 to 2.16)	1.61 (1.18 to 2.19)
Quartile 2	75 (57.3)	1.27 (0.93 to 1.73)	1.27 (0.93 to 1.73)	1.30 (0.95 to 1.78)
Quartile 3	82 (54.3)	1.19 (0.88 to 1.60)	1.21 (0.89 to 1.63)	1.26 (0.93 to 1.71)
Quartile 4	89 (47.3)	1.00	1.00	1.00
WC				
Quartile 1	58 (62.4)	1.00	1.00	1.00
Quartile 2	90 (52.6)	0.78 (0.56 to 1.09)	0.77 (0.55 to 1.08)	0.76 (0.55 to 1.07)
Quartile 3	92 (54.4)	0.77 (0.55 to 1.07)	0.76 (0.55 to 1.07)	0.75 (0.54 to 1.05)
Quartile 4	88 (52.1)	0.74 (0.53 to 1.03)	0.73 (0.52 to 1.02)	0.72 (0.52 to 1.01)
Baseline age < 90 years				
BMI				
Quartile 1	58 (36.0)	2.34 (1.54 to 3.54)	2.43 (1.59 to 3.72)	2.57 (1.66 to 3.98)
Quartile 2	49 (36.3)	2.29 (1.49 to 3.51)	2.35 (1.53 to 3.62)	2.54 (1.63 to 3.96)
Quartile 3	67 (38.5)	2.51 (1.68 to 3.76)	2.62 (1.74 to 3.93)	2.63 (1.75 to 3.97)
Quartile 4	37 (17.9)	1.00	1.00	1.00
WC				
Quartile 1	49 (35.8)	1.00	1.00	1.00
Quartile 2	56 (33.1)	0.82 (0.56 to 1.21)	0.86 (0.58 to 1.27)	0.84 (0.57 to 1.24)
Quartile 3	55 (32.5)	0.77 (0.52 to 1.14)	0.79 (0.53 to 1.17)	0.81 (0.54 to 1.20)
Quartile 4	51 (25.3)	0.59 (0.40 to 0.88)	0.59 (0.40 to 0.88)	0.59 (0.39 to 0.89)
Baseline age ≥ 90 years				
BMI				
Quartile 1	273 (63.6)	1.35 (1.11 to 1.63)	1.36 (1.12 to 1.65)	1.35 (1.12 to 1.64)
Quartile 2	211 (59.3)	1.16 (0.95 to 1.42)	1.17 (0.96 to 1.43)	1.18 (0.96 to 1.44)
Quartile 3	199 (57.2)	1.03 (0.84 to 1.26)	1.05 (0.86 to 1.29)	1.07 (0.87 to 1.32)
Quartile 4	177 (56.4)	1.00	1.00	1.00
WC				
Quartile 1	272 (64.6)	1.00	1.00	1.00
Quartile 2	215 (58.1)	0.81 (0.68 to 0.97)	0.82 (0.68 to 0.98)	0.82 (0.69 to 0.99)
Quartile 3	191 (57.7)	0.75 (0.62 to 0.91)	0.75 (0.62 to 0.90)	0.75 (0.62 to 0.91)
Quartile 4	182 (56.0)	0.72 (0.60 to 0.87)	0.71 (0.59 to 0.86)	0.73 (0.60 to 0.88)

*BMI: body mass index; WC: waist circumference. Model 1 was adjusted for age (not adjusted in subgroup analysis by baseline age), sex (not adjusted in subgroup analysis by sex), ethnicity, and residence. Model 2 was additionally adjusted for education level, marriage, smoking, drinking, economic independence, and living pattern. Model 3 was further adjusted for regular exercise, hypertension, diabetes, heart disease, stroke, and adequate medication. In the analyses of BMI, the *P* values for interaction were 0.83 and 0.13 for the sex subgroup and baseline age subgroup (<90 and ≥ 90 years), whereas in the analyses of WC, they were 0.44 and 0.56, respectively.

**Figure 2:**
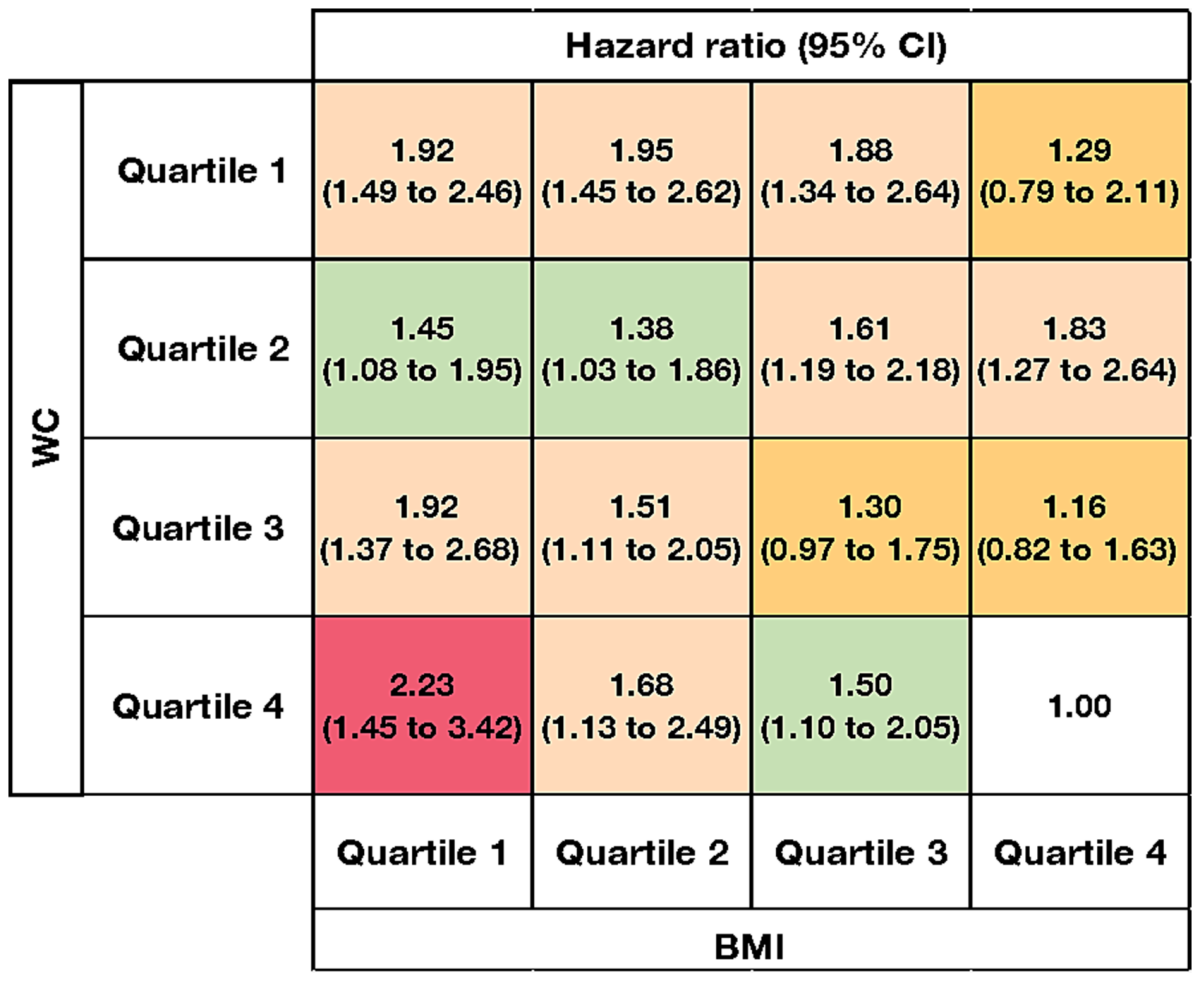
Joint association between body mass index, waist circumference, and all cause mortality in the oldest old with cognitive impairment. BMI: body mass index; WC: waist circumference. The orange color indicates that the association was not significant for the respective classification. The levels of hazard ratios were shown in green (1.00–1.50), peach puff (1.51–2.00), and pink (>2.00). Risk estimates were adjusted for age, sex, ethnicity, residence, education level, marriage, smoking, drinking, economic independence, living pattern, regular exercise, hypertension, diabetes, heart disease, stroke, and adequate medication. The blank cell with a value of 1.00 indicates the reference group.

The joint association between BMI and WC and all cause mortality were shown (Figure 2). We treated participants in the highest quartile of BMI and the highest quartile of WC as the reference group. The lowest hazard ratio was 1.00 in the reference group, whereas the highest hazard ratio was 2.23 (1.45 to 3.42) for those in the lowest quartile of BMI and highest quartile of WC. Overall, both lower quartiles of BMI and lower quartiles of WC were associated with higher mortality risk. For example, the hazard ratios for all-cause mortality were 1.92 (1.49 to 2.46) for quartile 1 of WC and quartile 1 of BMI, and 1.95 (1.45 to 2.62) for quartile 1 of WC and quartile 2 of BMI, both indicating a relatively higher mortality risk. In each quartile of WC, except for quartile 2, the hazard ratio for all-cause mortality decreased with increasing quartiles of BMI. Similarly, among participants in BMI quartiles 3 and 4, hazard ratios for all-cause mortality decreased as waist circumference quartiles increased.

## Discussion

We found J shaped association between BMI and all cause mortality among the cognitively impaired oldest old, with increased levels associated with reduced mortality risk. In addition, we observed a linear association pattern between WC and all cause mortality in the oldest old with cognitive impairment, with increased levels associated with low mortality risk. In addition, joint association analyses indicated that those both in the highest quartile of BMI and the highest quartile of WC had the lowest mortality risk.

### Comparison with other studies

Many prospective cohort studies have extensively investigated BMI in relation to all cause mortality.

Meta-analyses of prospective studies have found that higher BMI was associated with higher mortality risk (36, 37). However, the results were not consistent. A cohort study consisting of 4,968 participants aged 65 years or older reported that all-cause mortality risk was 11% lower in the overweight group (38). In the elderly population, Lv et al. indicated an opposite causal association between BMI and mortality (26). Among the population of cognitively impaired oldest old, the evidence linked BMI with all cause mortality is limited. One cohort study with data from the Swedish Dementia Registry demonstrated that higher BMI at the time of dementia diagnosis was associated with reduced mortality risk (16). Using the data in the National Health and Aging Trends Study with US participants aged 65 years and older, Natale et al. reported a negative relation between obesity and dementia-related mortality (17). In the report here, we found increased BMI associated with reduced mortality risk in the population of cognitively impaired oldest old, especially the J shaped association pattern. Both undernutrition and its risk are linked to higher mortality risk, independent of the cause of death (15), potentially through the effect of decreased BMI, which is in agreement with our findings. Our results were mostly consistent with previous results. Although a meta-analysis reported a U-shaped association between BMI and dementia-related mortality (39), we consider the different research populations and the various age phases of participants as potential explanations for this discrepancy. A cohort study with 776,571 participants in China observed that the average BMI was 24.4 kg/m^2^ in 2018 across the age group (40). It has to be noted that the mean level of BMI for the cognitively impaired oldest old in our study was lower (21.2 kg/m^2^) and our results should be interpreted on this basis. A cross-sectional cohort study with 16,538 participants aged 65 years or older also found that dementia caused weight loss (41).

In terms of abdominal obesity, several studies also have indicated that WC may be a better predictor than BMI for the risk of disease (42–44). In clinical practice, reducing WC is a critically important treatment target for decreasing adverse health risks across men and women (10). A study in Europe with 359,387 participants from nine countries found that the highest quintile of WC was associated with the highest mortality risk (6). Evidence indicates that undernutrition is associated with increased all cause mortality risk among hospitalized older adults, as well as those with ischemic stroke or frailty (45–47), presumably mediated by low levels of WC and BMI. Nutritional support appears to be associated with reduced mortality among undernourished patients, possibly by preventing further declines in BMI and WC or by improving these anthropometric measures (48). To our knowledge, our study was the first to explore the association between WC and all cause mortality in cognitively impaired older adults. Although Lv et al. reported a slight reduction in mortality risk with increasing levels of WC in the elderly population (26), our findings revealed a linear association pattern in the cognitively impaired oldest old, showing a remarkable reduction in mortality risk with increasing levels of WC. This might suggest that the cognitively impaired oldest old is distinct from the general elderly population. Our results from joint analyses indicated that the cognitively impaired oldest old with both higher BMI and higher WC was associated with lower mortality risk. The mechanism underlying the results is unknown. Several studies have demonstrated that a higher BMI may act as a protective factor in certain populations, a phenomenon often referred to as the “obesity paradox” or “reverse epidemiology” (49–53). This protection has been observed in older adults and patients with diseases such as heart failure, maintenance, and hemodialysis (49–54). Overall, the observed association patterns between BMI, WC, and all-cause mortality imply that overly strict reductions in BMI or WC could be detrimental in this population.

Strengths of this study include its largest sample size of the oldest old, high response rates for cognitive evaluation using the MMSE, and in-depth interviews assessing lifestyle variables. The present study has potential limitations. Firstly, considering the educational level and eyesight of the oldest old, we treated the missing values of the drawing item in the MMSE as a score of 0. However, we also excluded participants with missing values in this item and conducted a repeated analysis, which yielded almost the same association patterns. Secondly, although the waist-to-hip ratio is a commonly used indicator of abdominal obesity, we were unable to include it in our analysis due to the absence of hip circumference measurement. Thirdly, the current study included only CLHLS data which may limit the applicability of results to the general cognitively impaired oldest old.

## Conclusion

In the oldest old with cognitive impairment, we observed a J shaped association between BMI and all-cause mortality, with increased levels associated with reduced mortality risk. In addition, we also found a linear association between WC and all-cause mortality, with high levels associated with low mortality risk. In addition, joint analyses revealed that participants in the highest quartiles of both BMI and WC had the lowest mortality risk. The conclusion of this study implies that overly strict decreasing BMI or WC could be detrimental in this population.

## Data Availability

The original contributions presented in the study are included in the article/Supplementary material, further inquiries can be directed to the corresponding author.
