# Supplementary material for: Associations of body mass index and waist circumference with all cause mortality in the oldest old with cognitive impairment: a prospective cohort study
Source: Front Nutr. 2025 Jul 2;12:1561909. doi: 10.3389/fnut.2025.1561909 (PMC12263400; doi:10.3389/fnut.2025.1561909)
Supplement: Supplementary file 1 [file Data_Sheet_1.docx]

**Supplemental Online Content**

**Figure S1. Flow-chart of the study participants**

**Figure S2** **Associations of body mass index, waist circumference, and all cause mortality among the oldest old with cognitive impairment.**

**Table S1 Hazard ratios of all cause mortality according to BMI and WC stratified by cardiovascular disease in the oldest old with cognitive impairment.**

**Figure S1. Flow-chart of the study participants**

**The Chinese Longitudinal Healthy Longevity Survey (2011-2014), N=9765**

**nN**

**Excluded:**

Those with missing values in MMSE items: 377

Those with normal cognitive function based on MMSE scores: 6354

**Cognitively impaired participants, N=3034**

**Excluded:**

Those lost to follow-up: 228

Those with missing values in the measurement of body weight, body height, or waist circumference: 498

Those with three or more missing values in covariates: 122

Those with missing values in mortality date: 62

**Cognitively impaired participants, N=2124**

**
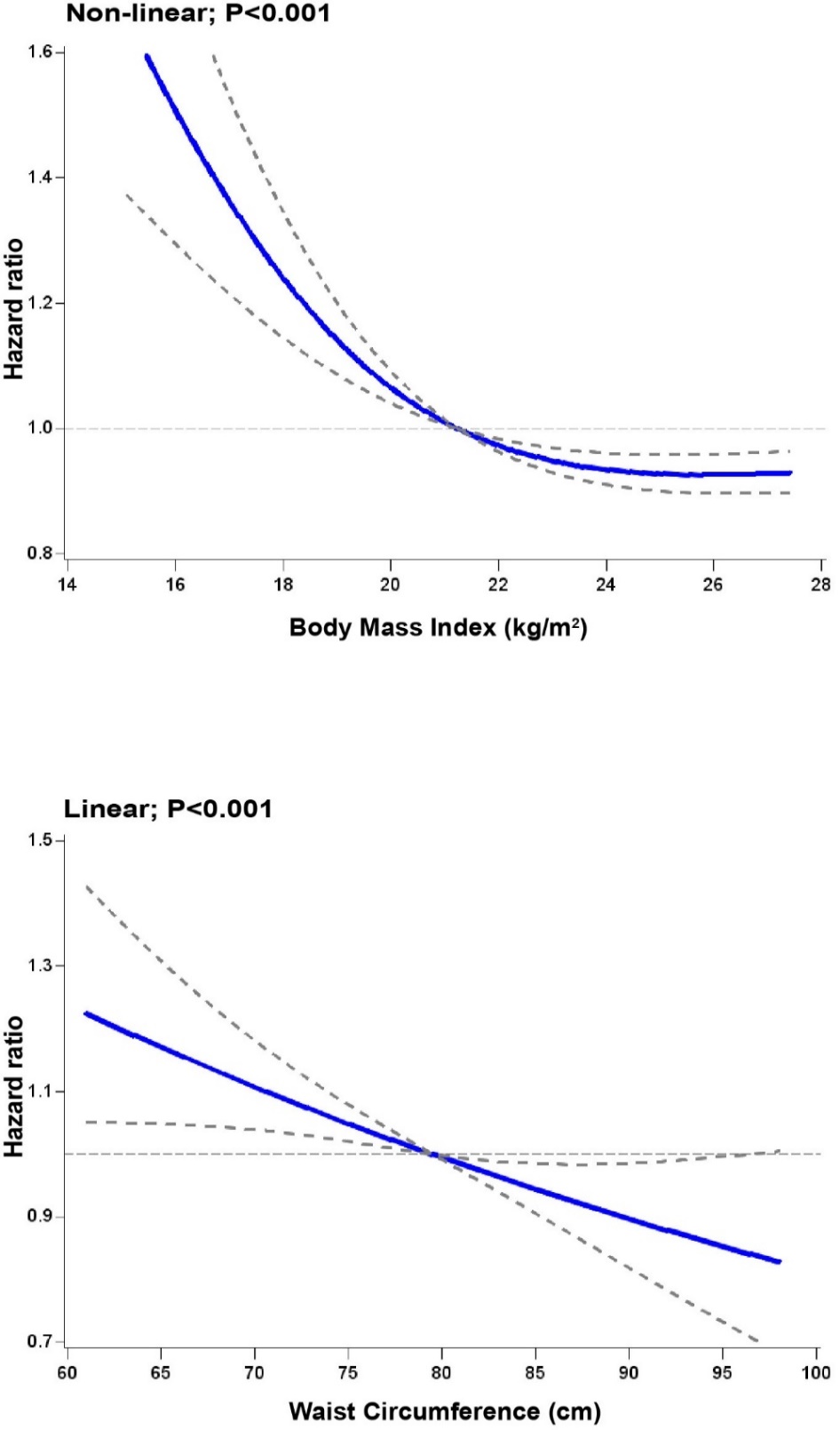
**

**Figure S2** **Associations of body mass index, waist circumference, and all cause mortality among the oldest old with cognitive impairment.** The hazard ratios were estimated for all cause mortality by levels of body mass index and waist circumference in cognitively impaired oldest old. Associations were investigated using restricted cubic spline with Cox proportional hazards models. Risk estimates were adjusted for age, sex, ethnicity, residence, education level, marriage, smoking, drinking, economic independence, living pattern, regular exercise, hypertension, diabetes, heart disease, stroke, and adequate medication. The units for BMI and WC are kg/m² and cm, respectively. The solid line is the calculated hazard ratio and the dashed line demonstrates 95% confidence intervals. P values for overall associations were all <0.001.

**Table S1 Hazard ratios of all cause mortality according to BMI and WC stratified by** **cardiovascular disease in the oldest old with cognitive impairment***

| **Variable** | **No (%) deaths** | **Model 1** | **Model 2** | **Model 3** |
| --- | --- | --- | --- | --- |
| **With cardiovascular disease** |  |  |  |  |
| BMI: |  |  |  |  |
| Quartile 1 | 49 (62.0) | 1.86 (1.23 to 2.81) | 1.93 (1.26 to 2.96) | 1.93 (1.26 to 2.96) |
| Quartile 2 | 44 (57.9) | 1.65 (1.07 to 2.53) | 1.76 (1.14 to 2.73) | 1.69 (1.09 to 2.64) |
| Quartile 3 | 44 (51.8) | 1.41 (0.92 to 2.16) | 1.48 (0.96 to 2.27) | 1.48 (0.96 to 2.28) |
| Quartile 4 | 44 (39.3) | 1.00 | 1.00 | 1.00 |
| WC: |  |  |  |  |
| Quartile 1 | 50 (61.7) | 1.00 | 1.00 | 1.00 |
| Quartile 2 | 37 (52.1) | 0.67 (0.43 to 1.04) | 0.63 (0.40 to 0.99) | 0.65 (0.41 to 1.02) |
| Quartile 3 | 42 (49.4) | 0.65 (0.43 to 0.98) | 0.63 (0.41 to 0.96) | 0.68 (0.44 to 1.04) |
| Quartile 4 | 52 (45.2) | 0.61 (0.41 to 0.91) | 0.58 (0.39 to 0.87) | 0.60 (0.40 to 0.92) |
| **Without cardiovascular disease** |  |  |  |  |
| BMI: |  |  |  |  |
| Quartile 1 | 282 (55.2) | 1.48 (1.22 to 1.79) | 1.46 (1.20 to 1.77) | 1.43 (1.18 to 1.74) |
| Quartile 2 | 216 (52.1) | 1.30 (1.06 to 1.59) | 1.28 (1.05 to 1.57) | 1.29 (1.05 to 1.59) |
| Quartile 3 | 222 (50.8) | 1.24 (1.01 to 1.51) | 1.25 (1.02 to 1.53) | 1.26 (1.03 to 1.54) |
| Quartile 4 | 170 (41.6) | 1.00 | 1.00 | 1.00 |
| WC: |  |  |  |  |
| Quartile 1 | 271 (56.8) | 1.00 | 1.00 | 1.00 |
| Quartile 2 | 234 (50.0) | 0.84 (0.71 to 1.00) | 0.85 (0.71 to 1.01) | 0.86 (0.72 to 1.02) |
| Quartile 3 | 204 (49.2) | 0.78 (0.65 to 0.94) | 0.78 (0.65 to 0.94) | 0.80 (0.66 to 0.96) |
| Quartile 4 | 181 (43.9) | 0.69 (0.57 to 0.84) | 0.70 (0.58 to 0.84) | 0.72 (0.59 to 0.87) |

*BMI: body mass index; WC: waist circumference.

Participants with stroke or heart disease were classified as having cardiovascular disease.

Model 1 was adjusted for age, sex, ethnicity, and residence. Model 2 was further adjusted for education level, marriage, smoking, drinking, economic independence, and living patterns. Model 3 was additionally adjusted for regular exercise, hypertension, diabetes, and adequate medication. In terms of BMI and WC, the P values for interaction in the cardiovascular disease subgroup were 0.16 and 0.69, respectively.
